# Left Atrial Remodeling after Mitral Valve Repair for Primary Mitral Regurgitation: Evolution over Time and Prognostic Significance

**DOI:** 10.3390/jcdd9070230

**Published:** 2022-07-18

**Authors:** Jan Stassen, Aniek L. van Wijngaarden, Hoi W. Wu, Meindert Palmen, Anton Tomsic, Victoria Delgado, Jeroen J. Bax, Nina Ajmone Marsan

**Affiliations:** 1 Department of Cardiology, Leiden University Medical Center, 2333 ZA Leiden, The Netherlands; j.stassen@lumc.nl (J.S.); a.l.van_wijngaarden@lumc.nl (A.L.v.W.); h.w.wu@lumc.nl (H.W.W.); v.delgado@lumc.nl (V.D.); j.j.bax@lumc.nl (J.J.B.); 2 Department of Cardiology, Jessa Hospital, 3500 Hasselt, Belgium; 3 Department of Thoracic Surgery, Leiden University Medical Center, 2333 ZA Leiden, The Netherlands; m.palmen@lumc.nl (M.P.); a.tomsic@lumc.nl (A.T.); 4 Department of Cardiology, Turku Heart Center, University of Turku and Turku University Hospital, 20521 Turku, Finland

**Keywords:** primary mitral regurgitation, left atrial remodeling, left atrium volume index, mitral valve repair, mortality

## Abstract

Left atrial (LA) dilatation is associated with worse outcomes in primary mitral regurgitation (MR). However, the effects of mitral valve repair on LA size and its prognostic implications are not well known. In the current study, LA volume index (LAVi) and LA reservoir strain (LASr) were evaluated immediately before and after surgery, and during long-term follow-up in 226 patients undergoing mitral valve repair for primary MR (age 62 ± 13 years, 66% male). Mean LAVi was reduced significantly after surgery and at long-term follow-up (from 56 ± 28 to 38 ± 21 to 32 ± 17 mL/m^2^; *p* < 0.001). LASr reduced significantly after surgery but increased again during the long-term (from 23.6 ± 9.4 to 11.5 ± 5.0 to 17.3 ± 7.5%; *p* < 0.001). Age, pre-operative LAVi, MR severity, and postoperative transmitral pressure gradient were associated with LA reverse remodeling by the long-term check-up. During a median follow-up of 72 (40–114) months, 43 (19%) patients died. Patients with LAVi ≥ 42 mL/m^2^ at long-term follow-up showed significant higher mortality rates compared to patients with LAVI < 42 mL/m^2^ (*p* < 0.001), even after adjusting for clinical covariates. In conclusion, significant LA reverse remodeling was observed both immediately and at long-term follow-up after mitral valve repair. LA dilatation at long term follow-up after surgery was still associated with all-cause mortality.

## 1. Introduction

Primary mitral regurgitation (MR) represents one of the most common valvular heart diseases and is associated with increased risks of morbidity and mortality when left untreated [1,2]. Surgical repair or replacement remains the only therapeutic strategy to treat patients with significant primary MR, and optimal timing of intervention is crucial to improve outcomes with minimal operative risk [3]. Currently, surgery is recommended in the presence of symptoms or left ventricular (LV) dilatation/dysfunction [4,5], although this is still associated with an increased risk of post-operative morbidity and mortality [6,7]. Primary MR also induces progressive left atrial (LA) dilatation and dysfunction [8], which have both been associated with worse outcomes in patients undergoing mitral valve (MV) surgery [9,10,11,12]. Therefore, current guidelines suggest that MV repair should also be considered in the presence of significant LA dilatation (i.e., LA volume index (LAVi) ≥ 60 mL/m^2^). Although the effect of MV repair on LV remodeling has been extensively studied [13,14], little is known about the effect of MV repair on LA size and function. Nonetheless, as LA dilatation has shown prognostic value before surgery, the extent of LA reverse remodeling after MV repair could also have important clinical implications. Therefore, the aim of the study was to: (1) evaluate the changes in LA size and function after MV repair, (2) identify correlates of improvement in LA size and function after MV repair, and (3) assess the prognostic significance of LA size after MV repair in patients with significant primary MR.

## 2. Methods

### 2.1. Patient Population

Patients who underwent MV repair for significant primary MR at the Leiden University Medical Center, The Netherlands, between 2000 and 2019 were identified. Patients with rheumatic valve disease, active endocarditis, connective tissue disorders, hypertrophic cardiomyopathy, congenital heart disease, previous cardiac surgery, significant mitral stenosis (defined as mean gradient > 5 mmHg), or significant (i.e., more than mild) aortic valve disease were excluded (*n* = 51). All patients underwent complete clinical and echocardiographic evaluation before MV surgery. Patient information was prospectively collected in the departmental cardiology information system (EPD-vision; Leiden University Medical Center, Leiden, The Netherlands) and retrospectively analyzed. Clinical data included demographic characteristics, cardiovascular risk factors, New York Heart Association functional class, and comorbidities. The surgical technique for MV repair has been previously described by our study group [15]. In summary, repair techniques included chordal replacement for anterior MV leaflet prolapse. Commissural prolapse was treated predominantly by papillary muscle head repositioning. For the posterior MV leaflet, a combination of resection and chordal replacement techniques was used. In all cases, a semi-rigid ring annuloplasty without downsizing was performed to stabilize the annulus and the suture line. Surgery was successful in all cases, with no residual MR ≥2 after intervention.

The study complies with the Declaration of Helsinki and was approved by the Institutional Review Board. Due to the retrospective design of this study, the Medical Ethical Committee waived the need for written informed consent.

### 2.2. Echocardiography

Standard transthoracic echocardiography was performed with commercially available ultrasound machines (Vivid 7 and E9, GE-Vingmed, Milwaukee, WI, USA). Electrocardiogram-triggered echocardiographic data were stored digitally in a cine-loop format for offline analysis using EchoPAC versions 113 and 203 (GE Medical Systems, Horten, Norway). LV end-diastolic and end-systolic diameters were measured from the parasternal long-axis view. LV volumes, LV ejection fraction, and LA volumes were measured using Simpson’s biplane method and indexed for body surface area [16]. MR severity was quantitatively assessed according to current recommendations using a multiparametric approach, including the effective regurgitant orifice area (using the proximal isovelocity surface area method), vena contracta, and regurgitant volume measurements, when feasible [17]. Systolic pulmonary artery pressure was estimated by measuring the maximal tricuspid regurgitant jet velocity with the simplified Bernoulli equation in combination with an estimation of the right atrial pressure, as recommended [16,18].

**Figure 1:**
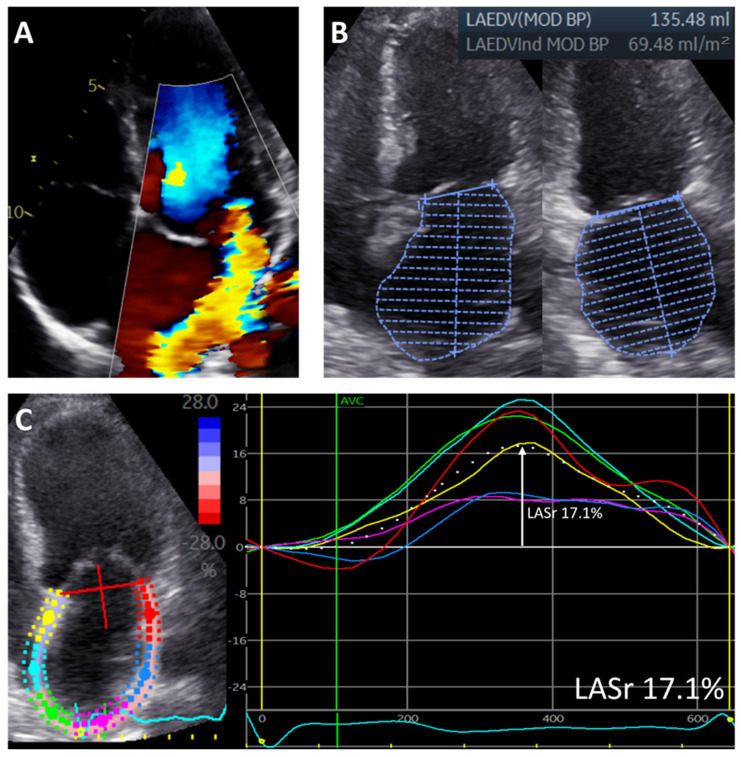
Measurements of left atrial volume and left atrial reservoir strain in a patient with significant primary mitral regurgitation. The figure shows a patient with significant primary MR (**A**) who had an enlarged LAVi (measured according to the biplane Simpson method) (**B**) and reduced LASr (measured as the peak value from the LA strain versus time curve derived from the 4-chamber view) (**C**). Regional strain curves are represented by different colors (yellow, red, blue, pink, green, light blue).

For the assessment of LA function, LA speckle-tracking strain was measured from the apical 4-chamber view with the onset of the QRS complex as the zero-reference point, according to current guidelines [19]. A region of interest was manually drawn along the LA endocardial border when LA was to its minimum volume after atrial contraction, excluding the pulmonary vein ostia and LA appendage. Automatic tracking of the LA wall by the software was visually verified and corrected by adjusting the region of interest or the width of the contour, ensuring appropriate capture of LA motion. LA reservoir strain (LASr) was then measured directly from the resulting LA strain versus time curve (Figure 1). To accurately assess the change in LA function over time, LASr was also adjusted for LAVi, since LA size changed significantly after MV surgery.

In each patient, echocardiography was evaluated at three different time points: pre-operatively (2 (0–5) months before the operation), immediately postoperatively (5 (4–6) days after the operation), and within 1–3 years as follow-up (on average 19 months (14–24) after the operation). These time intervals were chosen to separately assess the impact of MR reduction on LA volume reduction immediately after surgery and at long-term follow-up. To minimize the effects of other factors that could impact LA remodeling, patients who underwent MV reintervention, cardiac surgery. or myocardial infarction between the date of operation and date of final follow-up echocardiography were excluded from the study (*n* = 8). Patients were also excluded if LA strain analysis was not feasible due to inadequate quality of the images (*n* = 8).

### 2.3. Follow-Up

Patients were followed-up for the primary endpoint of all-cause mortality after the long-term follow-up echocardiography. Data on mortality were obtained from the Departmental of Cardiology’s information system (EPD-Vision, Leiden University Medical Center, Leiden, The Netherlands), which is linked to the governmental death registry database. Follow-up data were complete for all patients.

### 2.4. Statistical Analysis

Continuous variables are reported as mean ± SD when normally distributed and as median (interquartile range) when not normally distributed. Categorical variables are presented as absolute numbers and percentages. Continuous variables at different time points were evaluated using the paired samples *t*-test. Spearman correlation was used to evaluate the association between postoperative reduction in LAVi or LASr and clinical and echocardiographic variables. The intra- and interobserver variability of LASr and LAVi measurements were assessed by calculating the intra-class correlation coefficient in 20 randomly selected patients. For LASr, the intra-class correlation coefficients for inter -and intraobserver variability were 0.92 (95% CI: 0.84–0.97; *p* < 0.001) and 0.94 (95% CI: 0.85–0.98; *p* < 0.001). For LAVi, the intra-class correlation coefficients for inter -and intra-observer variability were 0.91 (95% CI: 0.86–0.96; *p* < 0.001) and 0.92 (95% CI: 0.84–0.95; *p* < 0.001). Furthermore, patients were divided into three groups according to the extent of LA dilatation, based on 2 cut-off points: 42 mL/m^2^ (based on the definition of mild versus moderate to severe dilated LA [16]) and 60 mL/m^2^ (based on current European guideline recommendations which recommend considering MV surgery in patients with LAVi ≥ 60 mL/m^2^ [20]). The three groups were: group 1—patients with preoperative LAVi < 42 mL/m^2^; group 2—patients with LAVi 42–59 mL/m^2^; and group 3—patients with LAVi ≥ 60 mL/m^2^. A general linear model for repeated measures was used to investigate changes in echocardiographic variables during follow-up between these three groups. Cumulative survival rates for all-cause mortality were estimated by the Kaplan–Meier method, and a log-rank test was used to compare groups. Cox proportional hazard regression analysis was performed to investigate the association between LAVi at long-term follow-up and all-cause mortality. The following covariables considered to have a potential prognostic impact and available in all patients at follow-up were included in the multivariable model: age, sex, coronary artery disease, and LV ejection fraction; the choice was limited by the number of events. The hazard ratio (HR) and 95% confidence (CI) were calculated and reported. All tests were two-sided, and *p*-values < 0.05 were considered statistically significant. Statistical analysis was performed using SPSS for Windows, version 25.0 (IBM Corporation, Armonk, NY, USA).

## 3. Results

### 3.1. Patient Population

A total of 226 patients (mean age 62 ± 13 years, 66% male) were included. Baseline clinical and echocardiographic characteristics are shown in Table 1. The mean preoperative LAVi was 56 ± 28 mL/m^2^ and a moderate to severe dilated LA (LAVi ≥ 42 mL/m^2^) was observed in 158 (69.9%) patients. Mean preoperative LASr was 23.6 ± 9.4%. Of interest, LV ejection fraction was on average preserved (65 ± 8%), and systolic pulmonary artery pressures were mostly within normal values (32 [25–45] mmHg).

### 3.2. Changes in LA Volume and Function after Mitral Valve Repair

**Figure 2:**
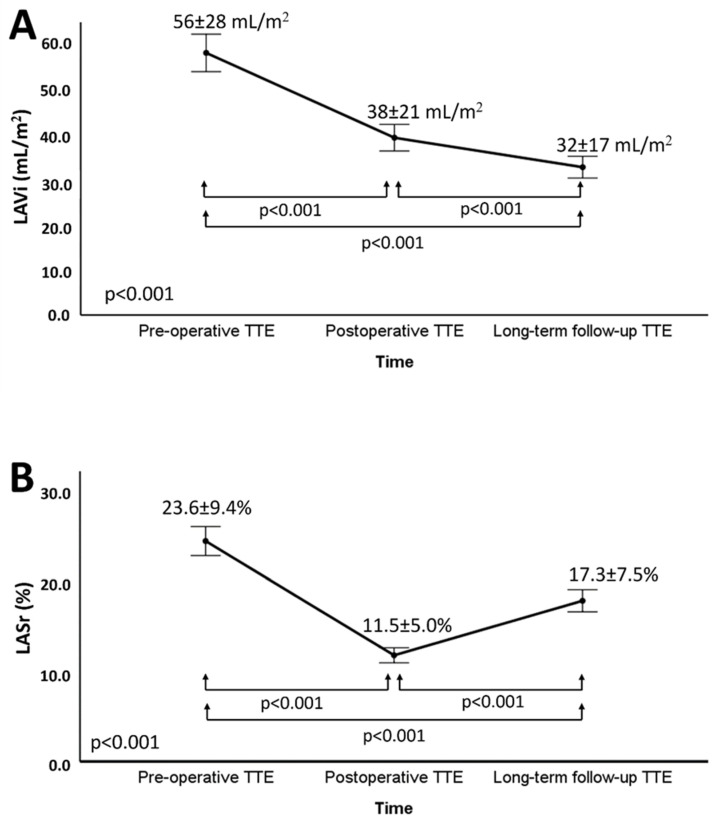
Changes in left atrium volume index (**A**) and left atrium reservoir strain (**B**) over time. Panel A shows the changes in LAVi among baseline, postoperative, and long-term follow-up values. Panel B shows the changes in LASr among baseline, postoperative, and long-term follow-up values. LASr = left atrial reservoir strain; LAVi = left atrium volume index; TTE = transthoracic echocardiography.

**Figure 3:**
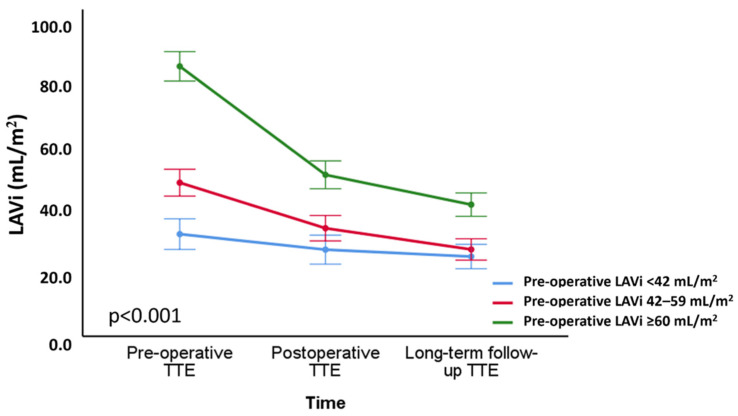
Changes in left atrium volume index over time according to the degree of preoperative left atrial dilatation. Changes in LAVi over time, according to three groups: patients with preoperativee LAVi < 42 mL/m^2^ (*n* = 68); patients with preoperative LAVi 42–59 mL/m^2^ (*n* = 88); patients with preoperative LAVi ≥ 60 mL/m^2^ (*n* = 70). LAVi = left atrium volume index; TTE = transthoracic echocardiography.

Mean LAVi reduced significantly immediately after surgery (from 56 ± 28 mL/m^2^ to 38 ± 21 mL/m^2^; *p* < 0.001) and further decreased during long-term follow-up (to 32 ± 17 mL/m^2^; *p* < 0.001) (Figure 2A). At the time of the long-term follow-up echocardiography, a LAVi ≥ 42 mL/m^2^ was still present only in 37 (16.4%) patients. Patients were further divided into three groups based on the preoperative LAVi: patients with preoperative LAVi < 42 mL/m^2^ (*n* = 68); patients with LAVi 42–59 mL/m^2^ (*n* = 88); patients with LAVi ≥ 60 mL/m^2^ (*n* = 70). The changes in LAVi over time among these groups are shown in Figure 3: although patients with a LAVi ≥ 60 mL/m^2^ at baseline showed the most pronounced reduction in LAVi, their volumes at long-term follow-up remained above the range of normality.

LASr (which was 23.6 ± 9.4% at baseline) reduced significantly immediately after surgery (to 11.5 ± 5.0%; *p* < 0.001) but increased again at long-term follow-up (to 17.3 ± 7.5%; *p* < 0.001) (Figure 2B). When corrected for LAVi, the ratio of LASr/LAVi (which was 0.55 ± 0.40% m^2^/mL at baseline) decreased significantly after surgery (to 0.36 ± 0.23% m^2^/mL; *p* < 0.001) but increased again at long-term follow-up to a value that was higher than the pre-operative value (to 0.69 ± 0.44% m^2^/mL; *p* < 0.001) (Figure S1).

The postoperative transmitral pressure gradient immediately after surgery was 2.6 (2.0–3.5) mmHg. No patients had residual MR ≥ 2 immediately after intervention, whereas 10 patients had severe and 19 patients had moderate MR at long-term follow-up. The changes in other echocardiographic variables during long-term follow-up after MV surgery are shown in Table 2.

### 3.3. Correlates of Changes in LA Volume and Function

Several clinical and echocardiographic variables were tested for their possible correlations with the extent of LAVi reduction and LASr evolution at long-term follow-up (Table 3). Significant correlations were found between reduction in LAVi at long-term follow-up and age (r = −0.139; *p* = 0.037), preoperative LV end-diastolic volume index (r = 0.199; *p* = 0.003), preoperative LAVi (r = 0.498; *p* <0.001), preoperative effective regurgitant orifice area (r = 0.205; *p* = 0.004), preoperative regurgitant volume (r = 0.222; *p* = 0.002), and postoperative transmitral mean pressure gradient (r = −0.124; *p* = 0.026). After entering significant correlates found by univariable analysis into a multivariable analysis, age (*p* = 0.009), preoperative LAVi (*p* < 0.001), and regurgitant volume (*p* = 0.017) remained independently associated with the extent of postoperative LAVi reduction. For LASr, significant correlations were found between changes in LASr at long-term follow-up and age (r = −0.364; *p* <0.001), eGFR (r = 0.236, *p* = 0.001), preoperative LAVi (r = −0.319; *p* < 0.001), and preoperative LASr (r = 0.569; *p* < 0.001). In multivariable analysis, only age (*p* = 0.029) and pre-operative LASr (*p* < 0.001) remained independently associated with change in postoperative LASr.

### 3.4. Outcome

**Figure 4:**
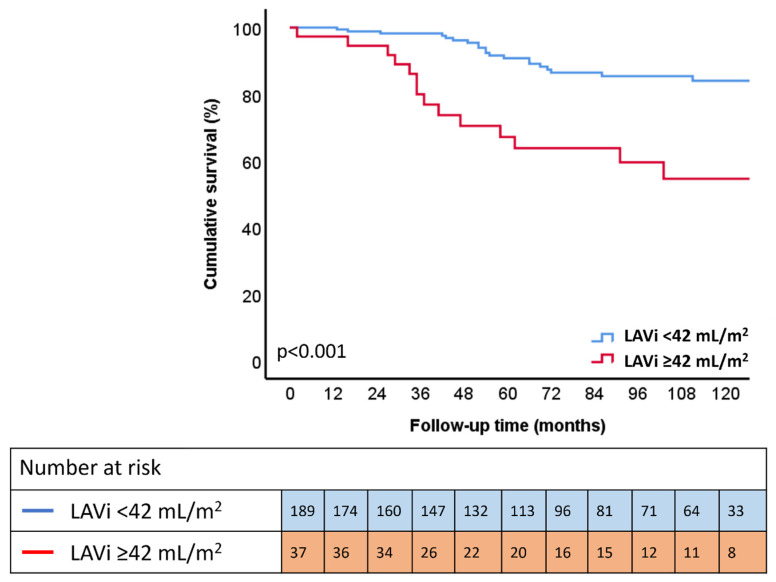
Kaplan–Meier survival curve for all-cause mortality according to LAVi at long-term follow-up. Time to all-cause mortality according to LAVi at long-term follow-up: LAVi < 42 mL/m^2^ (blue) and LAVi ≥ 42 mL/m^2^ (red).

During a median follow-up of 72 (40–114) months after the long-term follow-up echocardiography, 43 (19.0%) patients died. Patients who had LAVi ≥42 mL/m^2^ at long-term follow-up showed significant higher mortality rates compared to patients with LAVI < 42 mL/m^2^ (*p* < 0.001) (Figure 4). Univariable Cox regression analysis showed that long-term follow-up LAVi as a continuous variable (HR: 1.021; CI 1.012 to 1.031; *p* < 0.001) and categorical variable (LAVi ≥ 42 mL/m^2^) (HR: 3.729: CI 2.019 to 6.890; *p* < 0.001) was significantly associated with all-cause mortality (Table 4). Multivariable Cox regression analysis showed that, after adjusting for age, sex, and coronary artery disease, LAVi as a continuous variable (HR: 1.014; CI 1.011 to 1.027; *p* = 0.033) and a categorical variable (HR: 2.494; CI 1.292 to 4.815; *p* = 0.006) remained independently associated with all-cause mortality. In a sensitivity analysis, these findings were confirmed using a second multivariable model, adjusting for age, sex, and LV ejection fraction; and a third multivariable model, adjusting for age, sex, and atrial fibrillation (Table 4). Of interest, patients with more impaired LASr at long-term follow-up (when classified according to the median value of 17%) also showed significantly higher mortality rates on the Kaplan–Meier curve shown in Figure S2.

## 4. Discussion

The main results of the current study can be summarized as follows: (1) significant LA reverse remodeling was observed both immediately and at long-term follow-up after MV repair; (2) LA reverse remodeling at long-term follow-up was inversely correlated with age and postoperative transmitral pressure gradient; and positively correlated with preoperative LA and LV volumes, and preoperative MR severity; and (3) LA dilatation at long-term follow-up was still associated with all-cause mortality.

### 4.1. LA Reverse Remodeling after MV Repair

Currently, guidelines for the management of valvular heart disease strongly recommend MV repair for severe primary MR in the presence of symptoms or LV dysfunction [4,5]. However, surgery based on these indications is associated with worse short- and long-term outcomes when compared to early surgery [6]. Therefore, research is still focused on identifying prognostic parameters that may help to optimize timing of intervention in patients with primary MR. More recently, LA enlargement emerged as a strong prognostic parameter in different cardiovascular diseases, including MR, and is now an additional criterion for which MV repair should be considered according to European guidelines (but not American guidelines) [9,10,12]. Although the prognostic implications of preoperative LA size in patients undergoing MV repair are well-known, data on LA reverse remodeling after MV surgery remain largely unexplored. In a study including 79 patients with severe degenerative MR, LA dimensions significantly decreased within 1 to 6 months after surgery [21]. Similarly, in 65 patients with primary MR, Marsan and colleagues evaluated LA volumes with 3-dimensional echocardiography before and after MV surgery and demonstrated significant LA reverse remodeling when MV repair was performed at an early stage [22]. Our study expands on these results and shows that LA reverse remodeling is a continuous process. LA volume decreased significantly both immediately and again by long-term follow-up after MV surgery. The immediate volume reduction, which occurred only a few days after MV intervention, was probably a direct consequence of the elimination of the regurgitant volume, inducing a proportional decline in LAVi (“passive process”). In contrast, the progressive volume reduction at long-term follow-up could potentially be explained by a reduction in LA wall stress, facilitating LA myocardial reverse remodeling and enhancing LA contractility, thereby promoting a reduction in LA size (“active process”).

### 4.2. Predictors of LA Reverse Remodeling after MV Repair

The main correlates of postoperative LA volume reduction in the current study were: age, preoperative LA and LV volume, postoperative transmitral pressure gradient, and preoperative MR severity.

The current study therefore supports data from previous studies, demonstrating an important association between preoperative LA volume and the extent of LA reverse remodeling after surgery [21,22]. This implies that patients with pronounced LA enlargement still experience significant LA reverse remodeling after MV repair. However, although LAVi reduced to a normal-ranged value in the overall population, patients with a pre-operative LAVi > 60 mL/m^2^ still showed on average a dilated LAVi at long-term follow-up. Consequently, patients with pronounced LA dilatation probably already have irreversible LA structural changes and fibrosis, which prevents complete reverse remodeling to a normal-sized LA. This would also explain the correlation between higher preoperative LA volumes and lower values (impaired) of postoperative LASr, a parameter which has been associated with LA fibrosis [23]. These observations emphasize once more the need to carefully monitor LA volume when assessing patients with significant primary MR, and that lower preoperative cut-off values to refer patients for surgery should perhaps be considered to avoid irreversible LA damage.

Atrial reverse remodeling was also correlated with MR severity, a finding that can probably be explained by the immediate volume reduction due to the elimination of the regurgitant volume. However, patients with more severe MR also develop more atrial fibrosis, again potentially explaining the inverse correlation between MR severity and postoperative LASr which was seen in the current study. Additionally, postoperative transmitral pressure gradient was significantly associated with LA size/function, and the relationship between pressure overload and LA size/function has been previously demonstrated also in patients with pure mitral stenosis [24,25].

### 4.3. Prognostic Implications

Significant MR induces a volume overload on the LA, facilitating LA adverse remodeling. LA enlargement is known to be an important predictor of adverse cardiovascular events, including new onset atrial fibrillation, stroke, heart failure hospitalization, and death [26]. However, no data so far have been published on the prognostic value of LA size after MV repair, which has an impact on LA size and should therefore be taken into consideration. The current study shows that LA reverse remodeling is common after MV repair for significant primary MR, and most patients will have normalization of their LA size by long-term follow-up. However, patients with a very dilated preoperative LA still show still a dilated LA at follow-up, which is associated with worse overall survival. This observation supports the importance of a timely surgical approach in primary MR, but randomized trials are needed to confirm this hypothesis.

### 4.4. Limitations

This study was limited by the retrospective, observational design. It has also been performed in a tertiary referral center that is highly experienced in MV repair, and the results may therefore not be generalizable to other centers. Of note, information on ring-sizing was not available, which may have had an influence on the changes in LA size and function. Vendor-specific software was used, and this must be taken into consideration when assessing LA strain with different software. Additionally, LA volume was assessed with 2-dimensional echocardiography, which has its geometrical limitations when compared to 3-dimensional echocardiography. Although we excluded patients with major events during follow-up after MV repair, other non-reported changes (in medication or comorbidities) may still have influenced LA remodeling after surgery. In addition, data on heart failure hospitalization, atrial fibrillation, stroke, and cause of death after surgery were not available. Larger studies are needed to confirm the results of the present study and to demonstrate the prognostic value of a reduction in LA volume after surgery for primary MR.

## 5. Conclusions

LA reverse remodeling is common after MV repair for severe primary MR and is a continuous process. LA volume decreases both immediately and again by long-term follow-up after surgery. In addition, LA dilatation at long-term follow-up after surgery is still an important determinant of prognosis in these patients.

## Figures and Tables

**Table 1:** Baseline clinical and echocardiographic characteristics of the total study population.

	Study Population (*n* = 226)
Clinical characteristics	
Age, years	62.2 ± 12.6
Male sex	149 (65.9%)
Heart rate, bpm	76 ± 23
Systolic BP, mmHg	134 ± 20
Diastolic BP, mmHg	78 ± 11
BMI, kg/m^2^	25.2 ± 3.5
Arterial hypertension	86 (38.1%)
Diabetes mellitus	4 (1.8%)
(ex)smoker	69 (32.9%)
Coronary artery disease	44 (20.0%)
COPD	15 (6.8%)
eGFR, mL/min/1.73 m^2^	81.5 ± 25.2
CKD (eGFR < 60 mL/min/1.73 m^2^)	44 (19.6%)
Atrial fibrillation	75 (33.2%)
NYHA class ≥ II	165 (73.0%)
Mitral valve lesion and associated surgical procedures	
Prolapsing leaflet	
Anterior	26 (11.5%)
Posterior	161 (71.%2)
Both	39 (17.3%)
Associated surgical procedures	
CABG	35 (15.5%)
TVP	113 (50.0%)
MAZE	64 (28.3%)
LAA occlusion	30 (13.3%)
Echocardiographic characteristics	
LVEDD, mm	54.5 ± 6.6
LVESD, mm	33.4 ± 7.0
LVEDVi, mL/m^2^	71 ± 19
LVESVi, mL/m^2^	24 (19–31)
LVEF, %	65 ± 8
sPAP, mmHg	32 (25–45)
MR EROA, mm^2^	41 (29–55)
MR vena contracta, mm	7.2 ± 1.8
MR Rvol, mL	55 ± 23
LAVi, mL/m^2^	56 ± 28
LASr, %	23.6 ± 9.4

Values are presented as mean ± SD, median (IQR), or *n* (%). BMI = body mass index; BP = blood pressure; bpm = beats per minute; CABG = coronary artery bypass grafting; CKD = chronic kidney disease (defined as eGFR < 60 mL/min/1.73 m^2^); COPD = chronic obstructive pulmonary disease; eGFR = estimated glomerular filtration rate; EROA = effective regurgitant orifice area; LAA = left atrial appendage; LASr = left atrial reservoir strain; LAVi = left atrial volume index; LVEDD = left ventricular end diastolic diameter; LVEDVi = left ventricular end diastolic volume index; LVEF = left ventricular ejection fraction; LVESD = left ventricular end systolic diameter; LVESVi = left ventricular end systolic volume index; MR = mitral regurgitation; NYHA = New York Heart Association; Rvol = regurgitant volume; sPAP = systolic pulmonary artery pressure; TVP = tricuspid valve annuloplasty.

**Table 2:** Changes in echocardiographic variables at long-term follow-up after mitral valve repair.

	Pre-Operative	Long-Term Follow-Up	*p* Value
LVEDD, mm	54.5 ± 6.6	49.0 ± 6.3	<0.001
LVESD, mm	33.4 ± 7.0	35.6 ± 7.6	<0.001
LVEDVi mL/m^2^	71 ± 19	57 ± 17	<0.001
LVESVi, mL/m^2^	24 (19–31)	25 (18–31)	0.315
LVEF, %	65 ± 8	55 ± 11	<0.001
sPAP, mmHg	32 (25–45)	27 (23–33)	<0.001
LAVi, mL/m^2^	56 ± 28	32 ± 17	<0.001
LASr, %	23.6 ± 9.4	17.3 ± 7.5	<0.001

Values are presented as mean ± SD, median (IQR). Abbreviations as in Table 1.

**Table 3:** Univariate correlates of changes in LA volume and function at long-term follow-up after mitral valve repair.

	Change in LAVi at Long-Term Follow-Up		Change in LASr at Long-Term Follow-Up	
	Spearman’s Correlation Coefficient	*p* Value	Spearman’s Correlation Coefficient	*p* Value
Demographic characteristics				
Age, years	−0.139	0.037	−0.364	<0.001
BMI, kg/m^2^	−0.124	0.062	0.095	0.192
Systolic BP, mmHg	0.002	0.978	−0.102	0.173
Diastolic BP, mmHg	−0.057	0.402	−0.057	0.448
eGFR, mL/min/1.73 m^2^	0.062	0.358	0.236	0.001
Pre-operative AF	0.134	0.094	0.062	0.239
Echocardiographic characteristics				
Preoperative LVEDVi, mL/m^2^	0.199	0.003	0.016	0.826
Preoperative LVESVi, mL/m^2^	0.060	0.369	−0.040	0.585
Preoperative LVEF, %	0.069	0.302	0.109	0.137
Preoperative LAVi, mL/m^2^	0.498	<0.001	−0.319	<0.001
Preoperative LASr, %	0.008	0.904	0.569	<0.001
Preoperative EROA, mm^2^	0.205	0.004	0.003	0.967
Preoperative Rvol, ml	0.222	0.002	−0.171	0.030
Postoperative TMPG immediate after intervention, mmHg	−0.150	0.026	−0.068	0.361

AF = atrial fibrillation; BMI = body mass index; BP = blood pressure; eGFR = estimated glomerular filtration rate; EROA = effective regurgitant orifice area; LASr = left atrial reservoir strain; LAVi = left atrial volume index; LVEDVi = left ventricular end diastolic volume index; LVEF = left ventricular ejection fraction; LVESVi = left ventricular end systolic volume index; MR = mitral regurgitation; Rvol = regurgitant volume; TMPG = transmitral mean pressure gradient.

**Table 4:** Cox regression analysis for all-cause mortality, starting from the long-term follow-up echocardiography.

	Univariable Analysis		Multivariable Analysis Adjusting for Age, Sex, CAD		Multivariable Analysis Adjusting for Age, Sex, LVEF		Multivariable Analysis Adjusting for Age, Sex, AF	
	HR (95% CI)	*p* Value	HR (95% CI)	*p* Value	HR (95% CI)	*p* Value	HR (95% CI)	*p* Value
LAVi, mL/m^2^ (continuous)	1.021 (1.012–1.031)	<0.001	1.014 (1.001–1.027)	0.033	1.014 (1.003–1.024)	0.012	1.012 (1.001–1.023)	0.027
LAVi < 42 mL/m^2^	Reference		Reference		Reference		Reference	
LAVi ≥ 42 mL/m^2^	3.729 (2.019–6.890)	<0.001	2.494 (1.292–4.815)	0.006	2.518 (1.320–4.806)	0.005	2.468 (1.295–4.704)	0.006

AF = atrial fibrillation; CAD = coronary artery disease; CI = confidence interval; HR = hazard ratio; LAVi = left atrial volume index; LVEF = left ventricular ejection fraction.

## Data Availability

The data underlying this article will be shared on reasonable request to the corresponding author.

## Supplementary Materials

The following supporting information can be downloaded at: https://www.mdpi.com/article/10.3390/jcdd9070230/s1. Figure S1: Changes in left atrial reservoir strain over time, corrected for left atrial volume. Figure S2: Kaplan–Meier survival curve for all-cause mortality according to LASr at long-term follow-up.

## Abbreviations

LA	left atrial
LASr	left atrial reservoir strain
LAVi	left atrial volume index
LV	left ventricular
MR	mitral regurgitation
MV	mitral valve
