# Supplementary material for: Left Atrial Remodeling after Mitral Valve Repair for Primary Mitral Regurgitation: Evolution over Time and Prognostic Significance
Source: J Cardiovasc Dev Dis. 2022 Jul 18;9(7):230. doi: 10.3390/jcdd9070230 (PMC9320730; doi:10.3390/jcdd9070230)
Supplement: Supplementary file 1 [file jcdd-09-00230-s001.zip › jcdd-1788028-supplementary.pdf]

Journal of
Cardiovascular
Development and Disease

Supplementary Materials

Left Atrial Remodeling after Mitral Valve Repair for Primary Mitral Regurgitation: Evolution over Time and Prognostic Significance

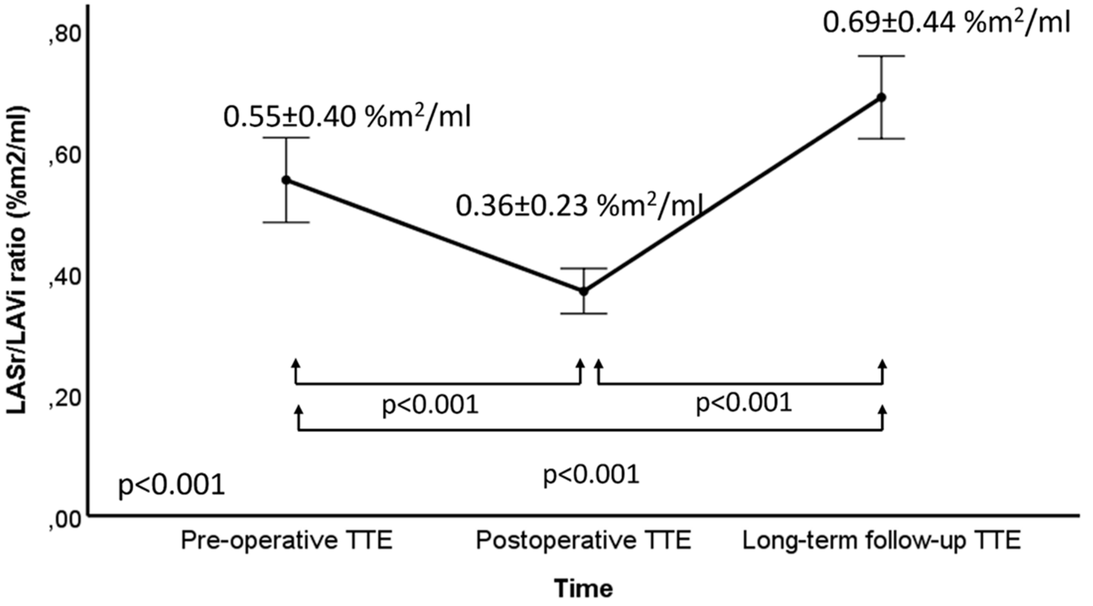

Figure S1. Changes in left atrial reservoir strain over time, corrected for left atrial volume.

This panel shows the changes in LASr, corrected for LAVi from baseline to postoperative -and long-term follow-up.

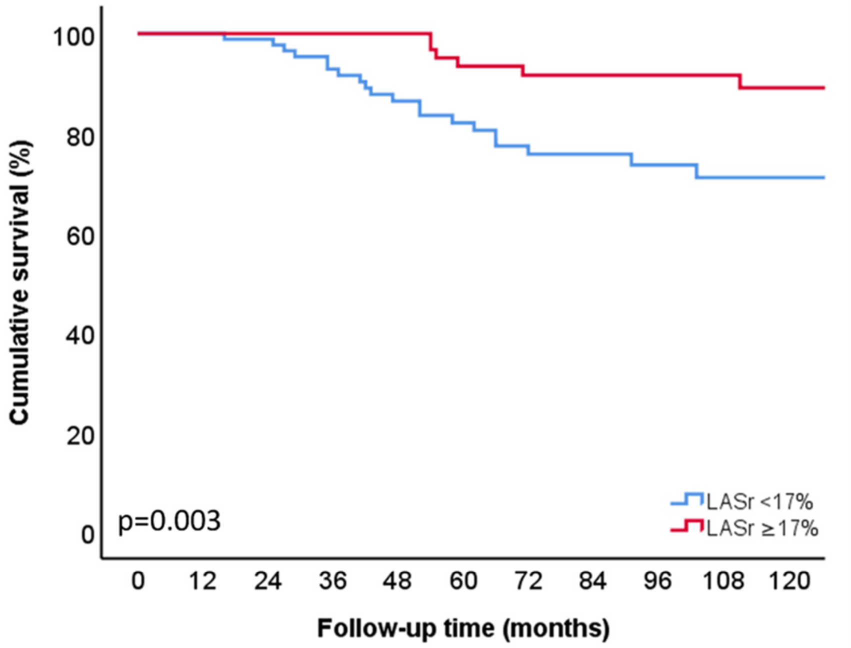

Number at risk											
LASr <17%	112	108	102	88	74	66	53	46	38	35	18
LASr ≥17%	114	102	92	85	80	67	59	50	45	40	23

Figure S2. Kaplan-Meier survival curve for all-cause mortality according to LASr at long-term follow-up.

Time to all-cause mortality according to LASr at long-term follow-up: LASr <17% (median value) (blue) and LASr ≥17% (red).

LASr = left atrial reservoir strain.
